# A semi-automated, isolation-free, high-throughput SARS-CoV-2 reverse transcriptase (RT) loop-mediated isothermal amplification (LAMP) test

**DOI:** 10.1038/s41598-021-00827-0

**Published:** 2021-11-01

**Authors:** Jonas Schmidt, Sandro Berghaus, Frithjof Blessing, Folker Wenzel, Holger Herbeck, Josef Blessing, Peter Schierack, Stefan Rödiger, Dirk Roggenbuck

**Affiliations:** 1 Institute for Laboratory Medicine, Singen, Germany; 2 Furtwangen University, Faculty of Medical and Life Sciences, Villingen-Schwenningen, Germany; 3 Institute of Biotechnology, Faculty Environment and Natural Sciences, Brandenburg University of Technology Cottbus-Senftenberg, Senftenberg, Germany; 4 Faculty of Health Sciences Brandenburg, Brandenburg University of Technology, Cottbus-Senftenberg, Universitätsplatz 1, 01968 Senftenberg, Germany

**Keywords:** Molecular biology, Viral infection

## Abstract

Shortages of reverse transcriptase (RT)-polymerase chain reaction (PCR) reagents and related equipment during the COVID-19 pandemic have demonstrated the need for alternative, high-throughput methods for severe acute respiratory syndrome coronavirus-2 (SARS-CoV-2)-mass screening in clinical diagnostic laboratories. A robust, SARS-CoV-2 RT-loop-mediated isothermal amplification (RT-LAMP) assay with high-throughput and short turnaround times in a clinical laboratory setting was established and compared to two conventional RT-PCR protocols using 323 samples of individuals with suspected SARS-CoV-2 infection. Limit of detection (LoD) and reproducibility of the isolation-free SARS-CoV-2 RT-LAMP test were determined. An almost perfect agreement (Cohen’s kappa > 0.8) between the novel test and two classical RT-PCR protocols with no systematic difference (McNemar’s test, P > 0.05) was observed. Sensitivity and specificity were in the range of 89.5 to 100% and 96.2 to 100% dependent on the reaction condition and the RT-PCR method used as reference. The isolation-free RT-LAMP assay showed high reproducibility (Tt intra-run coefficient of variation [CV] = 0.4%, Tt inter-run CV = 2.1%) with a LoD of 95 SARS-CoV-2 genome copies per reaction. The established SARS-CoV-2 RT-LAMP assay is a flexible and efficient alternative to conventional RT-PCR protocols, suitable for SARS-CoV-2 mass screening using existing laboratory infrastructure in clinical diagnostic laboratories.

## Introduction

Severe acute respiratory syndrome corona virus type 2 (SARS-CoV-2), an RNA virus that gives rise to coronavirus disease 2019 (COVID-19), has caused a major pandemic since it was first described in late 2019^1^. To better control and monitor the spread of COVID-19, the combined deployment of comprehensive surveillance, diagnostics, research, clinical treatment and vaccine development are required^2^.

Since SARS-CoV-2 is highly contagious, the main goal of laboratory diagnostics should be to identify infected individuals as quickly as possible. To accomplish this, amplification of viral nucleic acid plays a fundamental roll in assay strategies that have been established in many clinical diagnostic laboratories^2,3^. In general, the SARS-CoV-2 genome consists of 14 open reading frames (ORF) which involve possible targets for diagnostic nucleic acid-amplification assays^4,5^.

Besides reverse transcription (RT) polymerase chain reaction (PCR) (which is the gold standard method), isothermal amplification reactions are alternative techniques for nucleic acid detection^6,7^. Loop-mediated isothermal amplification (LAMP) has frequently been applied for SARS-CoV-2 detection^2,8,9^. With this technique, using four or six different target specific primers, and a *Bst* DNA polymerase with a high strand displacement activity, it is possible to detect viral nucleic acids with high sensitivity and specificity^10^. The main advantage of LAMP assays is the short isothermal reaction time (typically 10–25 min), which makes it faster and easier to perform compared to conventional RT-PCR^10^. There are many different read-out methods available including (real-time) fluorescence detection, colorimetric detection, turbidity, gel electrophoresis, and sequencing^10–12^. Such assays can therefore be applied under conditions where only basic laboratory equipment is available^13^.

To detect and estimate the amount of DNA in quantitative PCRs, the number of cycles is determined from which the threshold is significantly exceeded^14^. In contrast, isothermic amplification reactions are monocyclic. The amount of DNA is not determined by the number of cycles, instead it is determined by how much time is needed to exceed the background signal^15^. Since the duration of the cycle is generally constant, virtually any real-time PCR system can be used for the measurement. The specification of PCR-typical quantification points, such as the threshold cycle (Ct) or threshold time (Tt) value have been established in the literature.

Regarding SARS-CoV-2 diagnostics, most of the currently available LAMP protocols focus on qualitative colorimetric detection since interpretation is very easy by eye^9,16–19^. However, the temporary shortage of PCR supplies during the COVID-19 pandemic has demonstrated the need for alternatives to RT-PCR protocols, even in diagnostic laboratories with high throughput requirements and sophisticated laboratory equipment.

We have established a flexible and robust high-throughput SARS-CoV-2 RT-LAMP protocol, which can not only be used in combination with RNA isolation from nasopharyngeal swabs, but also with simple enzymatic digestion for sample preparation. To evaluate our protocol’s performance, we have compared it to two conventional RT-PCR protocols using clinical samples. To enable semi-automated high-throughput processing, we further established the protocol on a liquid handling station.

## Results

**Figure 1:**
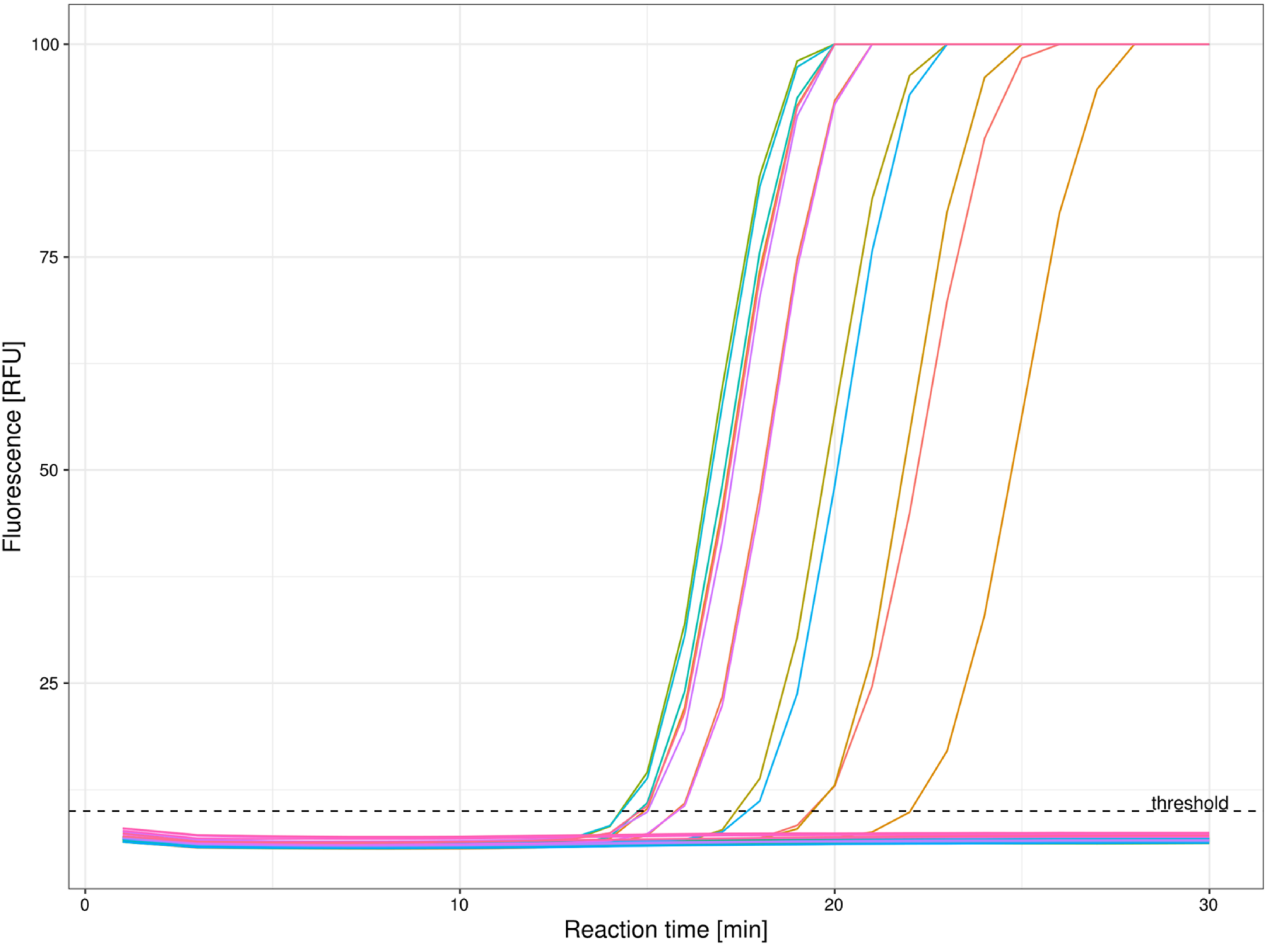
Amplification curves of SARS-CoV-2 RT-LAMP runs using an intercalating fluorescent dye for detection. During the 30 min isothermal incubation (65 °C), the fluorescence signal is read minutely on the FAM channel. A threshold is applied to identify positive samples and calculate the threshold time (Tt) value.

Applying the established SARS-CoV-2 RT-LAMP protocol, it was possible to distinguish positive and negative samples confirmed by standard RT-PCR test via fluorescence detection (Fig. 1). Positive samples showed a sigmoid increase of the fluorescence intensity over the isothermal incubation period. A banding pattern characteristic for LAMP reactions was observed by using conventional gel electrophoresis (Supplementary Fig. S1). The negative samples, as well as the no-template control, were below a predefined, operator adjustable threshold. The later can be used to calculate the Tt values of positive samples similar to calculation of Ct values in conventional RT-PCR. The external control targeting the human beta actin gene (ACTB) also showed a sigmoid increase in fluorescence intensity over time for all investigated clinical samples (Supplementary Fig. S2). The following method comparison experiments were performed without the additional external control reaction mix to increase throughput and efficiency.

After initial establishment, various method comparison experiments were performed using samples from individuals with suspected COVID-19 to compare the RT-LAMP assay with conventional RT-PCR protocols as standard methods for the detection of SARS-CoV-2 (Table 1).

**Table 1:** Method comparison of the SARS-CoV-2 RT-LAMP with RNA isolation with (I) the in-house RT-PCR protocol, (II) the commercial Labsystems RT-PCR kit and (III) the in-house RT-PCR using a liquid handler. Further, the method comparison between the isolation-free protocol of the RT-LAMP and the in-house RT-PCR is shown.

	RT-LAMP (isolation)—in-house RT-PCR	RT-LAMP (isolation)—Labsystems RT-PCR	RT-LAMP (isolation)—in-house RT-PCR (liquid handler)	RT-LAMP (isolation-free)—in-house RT-PCR
Sample size	70	70	188	65
True positive	34	34	22	39
True negative	34	32	166	25
False positive	0	0	0	1
False negative	2	4	0	0
Cohen’s Kappa (κ)	0.94	0.89	1	0.97
McNemar’s test (P)	0.55	0.22	NA^a^	0.79
Sensitivity (%) (95% CI)^b^	94.4 (81.3–99.3)	89.5 (75.2–97.1)	100 (84.6–100)	100 (91.0–100)
Specificity (%) (95% CI)^b^	100 (89.7–100)	100 (89.1–100)	100 (97.8–100)	96.2 (80.4–99.9)

^a^Not available. McNemar’s test cannot be calculated for perfect agreement.

^b^Confidence interval.

Using 70 isolated RNA samples, the RT-LAMP protocol was compared with our in-house standard RT-PCR protocol targeting the E gene, and with a commercial PCR kit targeting the E, N and ORF1ab genes. An almost perfect agreement (Cohen’s kappa [κ] > 0.8) between the RT-PCR protocols and the RT-LAMP protocol was observed with no systematic difference (McNemar’s test, P > 0.05). Sensitivity was 94.4% (95% confidence interval [CI] 81.3–99.3%) when using the in-house RT-PCR as a reference, and 89.5% (CI 75.2–97.1%) for the commercial RT-PCR kit as a reference. Specificity was 100% in both cases as no false positive results were observed.

**Figure 2:**
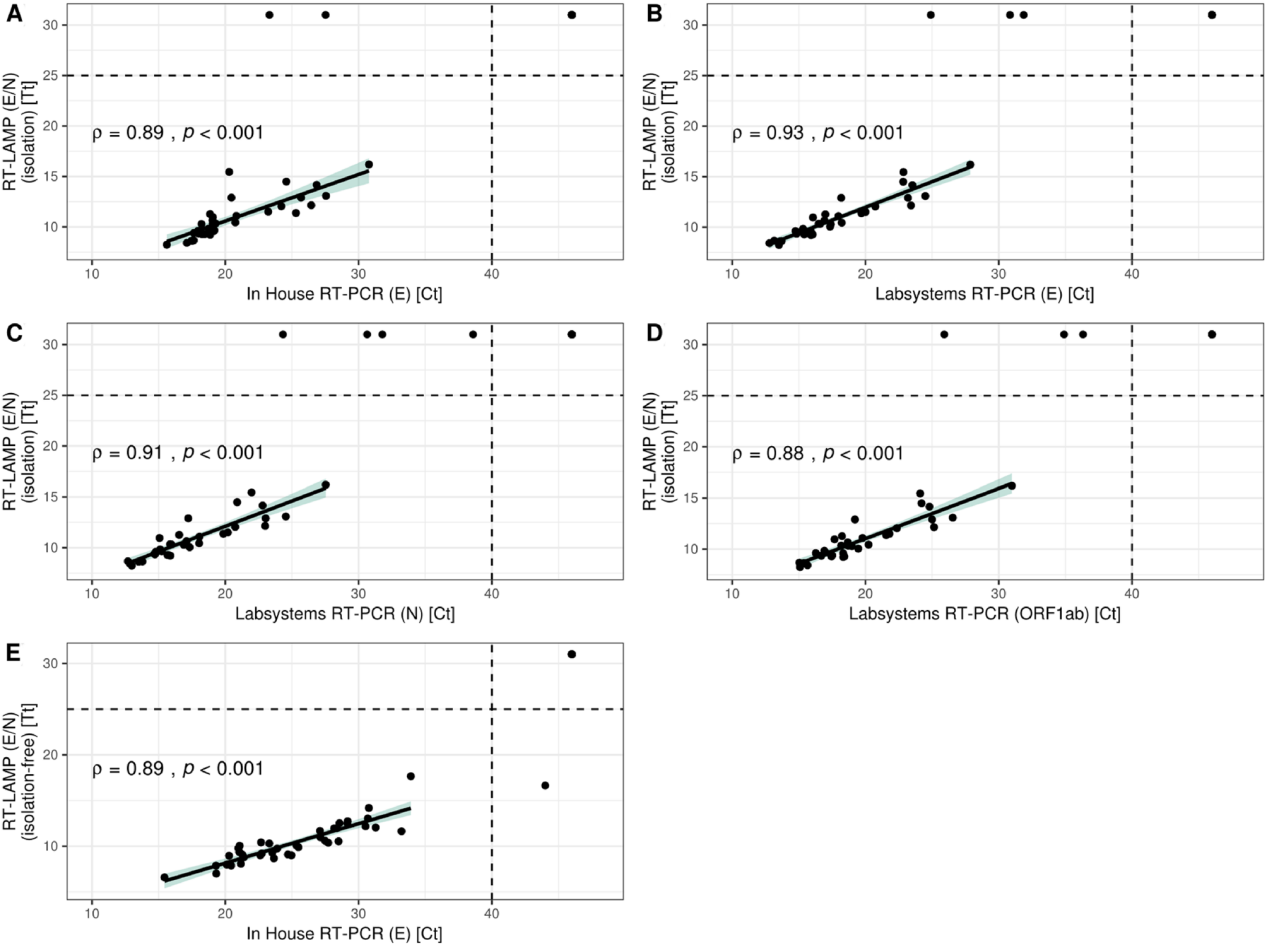
Comparison of the SARS-CoV-2 RT-LAMP Tt values and the RT-PCR Ct values. The dashed lines represent the negative cut-offs. Artificial Ct and Tt values above these cut-offs were assigned to negative samples for data visualization purpose. Spearman correlation results are shown. Only true positive samples were included into the correlation analysis. (**A**) SARS-CoV-2 RT-LAMP with RNA isolation compared to the in-house RT-PCR. (**B**) SARS-CoV-2 RT-LAMP with RNA isolation compared to the LabsystemsDx RT-PCR (E gene target). (**C**) SARS-CoV-2 RT-LAMP with RNA isolation compared to the LabsystemsDx RT-PCR (N gene target). (**D**) SARS-CoV-2 RT-LAMP with RNA isolation compared to the LabsystemsDx RT-PCR (ORF1ab gene target). (**E**) SARS-CoV-2 RT-LAMP without RNA isolation compared to the in-house RT-PCR.

The Tt values of the RT-LAMP assay showed a significant positive correlation with the RT-PCR data for all different targets (Rho [ϕ] > 0.8, P < 0.001) (Fig. 2A–D). Samples classified as false-negative (2/70) by the RT-LAMP test had Ct values in the range of 20–30 in the in-house RT-PCR. The Ct values of the samples classified as false-negative (4/70) in comparison with the commercial RT-PCR kit were in the range of 20–30 in one case, and above 30 in two cases. The remaining false-negative sample showed only a signal for the N gene target with a Ct value above 35 in the commercial RT-PCR.

In order to increase sample throughput, and decrease manual hands-on time, we adapted the SARS-CoV-2 RT-LAMP workflow on a liquid handling system. To evaluate the performance a method comparison to the in-house RT-PCR was performed using 188 isolated RNA samples. A perfect agreement (κ = 1) between the two methods was observed resulting in 100% (CI 84.6–100%) sensitivity and 100% (CI 97.8–100%) specificity of the SARS-CoV-2 RT-LAMP (Table 1).

To reduce the processing time further, an RNA isolation-free sample preparation step for nasopharyngeal swabs using proteinase K digestion was added to the RT-LAMP protocol. A method comparison to the in-house RT-PCR was carried out using 65 nasopharyngeal swabs. Near perfect agreement (κ = 0.97) was observed between both methods with no systematic difference (McNemar’s test, P = 0.79) (Table 1). Of the 65, a single sample was classified false-positive by the RT-LAMP assay with a Tt value above 15. A significant positive correlation of the RT-LAMP Tt values and the RT-PCR Ct values was observed (ϕ = 0.89, P < 0.001) (Fig. 2E). Sensitivity and specificity of the isolation-free RT-LAMP protocol compared to the in-house RT-PCR protocol were 100% (CI 91.0–100%) and 96.2% (CI 80.4–99.9%), respectively.

Using reference material with a known amount of SARS-CoV-2 genome copies and pools of positive and negative samples, performance characteristics of the isolation-free SARS-CoV-2 RT-LAMP protocol, including LoD and intra-/inter-assay reproducibility, were assessed. The LoD was determined at a concentration of 100,000 copies/ml. This equals 5000 copies in the eluate from a nasopharyngeal swab, or 95 copies in the RT-LAMP reaction (Supplementary Table S1). During the reproducibility runs, all positive and negative pool samples were correctly qualitatively assigned resulting in 100% intra-run and inter-run reproducibility. The intra-run median Tt value (10.81) of the positive sample pool was comparable to the inter-run median Tt value (10.71). As expected, the inter-run Tt variability was slightly higher (standard deviation [SD] 0.222, coefficient of variation [CV] 2.1%) compared to the intra-run Tt variability (SD 0.042, CV 0.4%) (Table 2).

**Table 2:** Intra- and inter-run reproducibility of the isolation-free SARS-CoV-2 RT-LAMP protocol. To determine the reproducibility a positive pool sample and a negative pool sample was measured in five replicates on three consecutive days.

	Intra-run reproducibility	Inter-run reproducibility
Qualitative agreement (%)	100	100
Median Tt value^a^	10.81	10.71
Standard deviation (SD)^a^	0.04	0.22
Coefficient of variation (CV) (%)^a^	0.4	2.1

^a^Median Tt value, standard deviation and the coefficient of variation are calculated based on the results from the positive pool.

## Discussion

Due to supply bottlenecks of PCR reagents, laboratory equipment, and reagents needed for RNA isolation from nasopharyngeal swabs, there have been major difficulties in SARS-CoV-2 routine diagnostic workflows during the recent SARS-CoV-2 pandemic. Therefore, we have established an RT-LAMP protocol for detection of SARS-CoV-2 which can be used as an alternative to conventional RT-PCR protocols. Using fluorescence as read-out, the protocol can be applied on conventional RT-PCR cyclers which are available in most clinical diagnostic laboratories. In addition, this detection method can be easily adapted to automated processing with standard workflows and has the potential to allow a quantitative analysis if needed. To further increase throughput and decrease hands-on time, we successfully adapted the SARS-CoV-2 RT-LAMP protocol on a liquid handling station. This has not only decreased sample processing time, but also reduces the risk of error due to manual processing. To further increase the robustness of the SARS-CoV-2 RT-LAMP assay, we combined the RT-LAMP protocol with a simple RNA isolation-free sample preparation using proteinase K digestion. A similar approach has been described for the preparation of saliva samples by Vogels et al.^20^. They demonstrated that saliva is a valid alternative to nasopharyngeal swabs with respect to SARS-CoV-2 testing and their protocol has received Emergency Use Authorization (EUA) from the U.S. Food and Drug Administration (FDA)^20^.

Since the beginning of the pandemic, the RT-LAMP technology has emerged as an additional tool for laboratory diagnosis of SARS-CoV-2 and COVID-19, in parallel with conventional RT-PCR^2^. Commercial test kits are already offered by different companies including Color Genomics, Lucira Health, and New England Biolabs. There is a high number of published RT-LAMP protocols available describing the detection of SARS-CoV-2 in various clinical specimens including nasopharyngeal swab extractions and saliva. In a point-of-care-test (POCT) setting, detection methods utilising visual inspection of the results are frequently used^21–26^. For application in a dedicated diagnostic laboratory environment there are different protocols available using fluorescence detection, or absorbance readings as quantitative measurement^27–33^. These advanced detection methods are also possible in POCT diagnostics and several protocols are available^34–36^.

By reducing the complexity of the sample preparation, processing time and therefore cost per reaction is reduced. Both aspects are important to consider when adapting assays for SARS-CoV-2 mass screening in clinical diagnostic laboratories. With the isolation-free RT-LAMP protocol, it is possible to process samples in less than 90 min, which is significantly faster than conventional RT-PCR protocols^8^.

During the initial phase of method establishment, an additional reaction mix per sample targeting human ACTB was included as an external control to account for sample quality. Since the results were positive for all investigated clinical samples, we discontinued using the external control during the following method comparison experiments to increase throughput and efficiency. For future experiments, to increase confidence in negative results while retaining high throughput and efficiency, it would be favourable to amplify and detect a human target as internal control in addition to the SARS-CoV-2 targets in a one-tube reaction using target specific probes^10,11^. This would increase the difficulty of the experimental design and execution but would also expand the overall analytical quality^10^.

To compare our RT-LAMP protocols with conventional RT-PCR protocols, we performed various method comparison experiments with clinical samples using an in-house RT-PCR assay as well as a commercial assay as standard. Under all conditions tested, the qualitative results of the RT-LAMP showed near perfect agreement with the RT-PCR assays, with no indication of a systematic difference (κ > 0.8, McNemar’s test, P > 0.05). Sensitivity ranged from 89.5% to 100% and specificity from 96.2 to 100%. This is consistent with the general observation that RT-LAMP assays show a reduced sensitivity compared to RT-PCR^8,16,37^. In our study, RT-PCR Ct values of the samples classified as false-negative by RT-LAMP were in a high range above 30 in most cases. This suggests that it is largely samples with a low viral load that are not detected by RT-LAMP. This could be due to the higher LoD compared to RT-PCR.

The isolation-free SARS-CoV-2 RT-LAMP test showed a LoD of 100,000 copies/ml or 95 copies per reaction which is consistent with data reported elsewhere^16,38^. Using positive and negative sample pools, our isolation-free RT-LAMP protocol was highly reproducible with no false classification over three runs with five replicates of each pool. The Tt values of the positive sample pool showed little variability in terms of both intra- and inter-run variability.

Using the dynamic pattern of viral load kinetics, Larremore et al. demonstrated that effective SARS-CoV-2 population screening depends primarily on the frequency of testing and speed of reporting^8^. Their study showed that test sensitivity was secondary, supporting RT-LAMP as a useful alternative or addition to RT-PCR despite the higher LoD.

Compared to the use of isolated RNA samples for SARS-CoV-2 detection, the isolation-free RT-LAMP assay showed a slightly reduced specificity of 96.2%. The sample, which was classified false positive by RT-LAMP, demonstrated a high Tt value above 15. Since there is no RNA purification step in the isolation-free protocol, the input sample matrix is more complex in the RT-LAMP reaction under these conditions. This could cause a slightly reduced specificity compared to an assay with RNA isolation. However, laboratory findings should always be interpreted alongside patient clinical history, thus a slightly reduced specificity should be acceptable.

Overall, the RT-LAMP Tt values showed a significant positive correlation with the RT-PCR Ct values. This is a valuable finding since both methods are based on the amplification of viral genetic material in the samples and apply an identical procedure for Ct value/Tt value calculation. As outlined by Engelmann et al., care must be taken when interpreting Ct or Tt values in a clinical setting with regard to viral infectivity since they could be biased by preanalytical issues and there is only little analytical test harmonization available^39^.

Although RT-LAMP has proven to be a fast and efficient alternative for SARS-CoV-2 screening in clinical diagnostic laboratories, there are some limitations. In addition to reduced analytical and diagnostic sensitivity, RT-LAMP assays carry a high risk for carry-over contamination due to an extremely high efficient reaction, which can lead to false positive results^10^. To reduce this risk it is important to take several precautions. Regarding assay design, we added dUTP/UDG to the reaction mixture to reduce the risk of cross contamination from previous runs. Further risk reduction can be achieved by spatial separation of sample preparation, master mix set up, RT-LAMP reaction set up and incubation/detection^10^. It is also fundamentally important that the reaction tubes are not opened after the LAMP reaction is complete^10^. The tubes should be tightly sealed and discarded immediately. Prior to the use of a RT-LAMP protocol in a clinical diagnostic laboratory, it is important to perform a comprehensive method validation in order to identify pitfalls and take appropriated measures.

SARS-CoV-2 screening is only one possible application in a clinical diagnostic laboratory. As RT-LAMP protocols are quite flexible this technique could be used more widely after the SARS-CoV-2 pandemic. Appropriate protocols have already been established, for Influenza virus, Zika virus, Dengue virus, Hepatitis C, respiratory syncytial virus, and *Streptococcus pneumoniae*^10,40–43^.

The measurement method is not bound to a technology, but can be used with different systems. The same applies to the evaluation of the data. In this study we used the software of the manufacturer. However, this can also be done with alternative open source software, as described by us^6,15,44^.

To summarise, we have established and evaluated a flexible SARS-CoV-2 RT-LAMP protocol which shows acceptable analytical performance and could be used as an alternative to RT-PCR in clinical diagnostic laboratories. Most currently available RT-LAMP protocols are focused on POCT approaches. Our goal was to optimise throughput and turnaround time based on already existing laboratory infrastructure in order to provide an assay which can be easily adapted to different conditions, and is suitable for SARS-CoV-2 mass screening.

## Materials and methods

### Clinical samples

A total of 323 nasopharyngeal swabs from individuals with suspected COVID-19 sent to the laboratory of the Institute for Laboratory Medicine (Singen, Germany) for SARS-CoV-2 screening were used for method comparison. All samples used were pseudo-anonymized surplus material from routine diagnostics and were retrieved from the laboratory’s sample storage only after initial testing was finished. No personal or medical patient data were recorded or analysed. All individuals gave their informed consent. The study has complied with all the relevant national regulations and institutional policies and has been approved by the ethics committee of the Brandenburg University of Technology Cottbus-Senftenberg (EK2020-16).

### Sample preparation and SARS-CoV-2 RT-LAMP protocol

During method development, the SARS-CoV-2 RT-LAMP assay was run with and without RNA isolation. RNA isolation from nasopharyngeal swabs was performed on chemagic 360 instruments (PerkinElmer, Waltham, USA) using PrepitoViral DNA/RNA300 isolation kits (PerkinElmer, Waltham, USA).

The isolation free workflow was done using proteinase K digestion^20^. All sample handling steps were performed under a biosafety cabinet class II. The nasopharyngeal swabs were flushed with 500 µl NaCl -solution (0.9%) by vortexing. 2.5 µl proteinase K solution (50 mg/ml) (ThermoFisher Scientific, Waltham, USA) was prepared in PCR reaction tubes and 50 µl of the sample eluate was added. After vortexing for 1 min, samples were incubated in a thermocycler (ThermoFisher Scientific, Waltham, USA) at 57 °C for 5 min followed by 95 °C for 5 min. After proteinase K digestion the samples were directly analysed by RT-LAMP.

A LAMP primer mix in nuclease free water targeting the N and E gene of SARS-CoV-2 (Supplementary Table S2) was used in combination with a WarmStart RT-LAMP kit including a fluorescent intercalating marker (New England Biolabs, Ipswich, USA)^45^. Individual primers were purchased from IDT (Integrated DNA Technologies, Coralville, USA). Deoxyuridine triphosphate (dUTP) and uracil-DNA glycosylase (UDG) (New England Biolabs, Ipswich, USA) were added to the reaction mix to prevent cross contamination from previous runs. WarmStart RTx Reverse Transcriptase (New England Biolabs, Ipswich, USA) was added to increase RT efficiency. To account for sample quality during the initial method establishment, a separate second reaction mix using a LAMP primer set targeting the human beta actin gene (ACTB) (Supplementary Table S3) was set up per sample as external control^45^.

A master mix was prepared and added to PCR reaction strips (Table 3). After addition of 1 µl RNA template, the strips were tightly sealed, gently mixed, and incubated for 5 min at 25 °C, and then for 30 min at 65 °C in a Rotor-Gene Q device (Qiagen, Hilden, Germany) or a LightCycler 96 (Roche, Basel, Switzerland). Fluorescence readings were taken every minute during this incubation period on the FAM channel as Bst polymerase is mainly active at 65 °C. A no-template control and a positive control (INSTAND, Düsseldorf, Germany) were added for all runs.

**Table 3:** Composition of the SARS-CoV-2 RT-LAMP reaction mix. Deoxyuridine triphosphate (dUTP) and Antarctic thermolabile Uracil DNA glycosylase (UDG) were applied to prevent cross-over contamination. Additional WarmStart reverse transcriptase was added to increase reverse transcription efficiency. During the initial method establishment, a second reactions mix was set up per sample using a LAMP primer mix targeting the human beta actin gene (ACTB) instead of SARS-CoV-2 as an external control to account for sample quality.

Reagent	Vendor	Volume per reaction (µl)
WarmStart LAMP Master Mix (2X)	New England Biolabs	12.5
Fluorescent dye (50X)	New England Biolabs	0.5
SARS LAMP Primer Mix (×10)/ACTB LAMP Primer Mix (10X)	Integrated DNA Technologies	2.5
H2O	VWR	7.625
dUTP	New England Biolabs	0.175
UDG	New England Biolabs	0.2
WarmStart RTX Reverse Transcriptase	New England Biolabs	0.5

### RT-PCR protocols

An in-house SARS-CoV-2 RT-PCR protocol and a commercial RT-PCR kit were used for method comparison. Since both assays work only with purified RNA, the above-mentioned RNA isolation procedure was mandatory prior to PCR testing.

The in-house PCR protocol was based on the QuantiTect Probe RT-PCR Kit (Qiagen, Hilden, Germany) with primers and a hydrolysis probe (Biomers, Ulm, Germany) targeting the E gene. Detection was done on the FAM channel of a LightCycler 96 instrument (Roche, Basel, Switzerland). Primer sequences and the temperature profile are shown in the appendix (Supplementary Information 1).

For comparison of the SARS-CoV-2 RT-LAMP protocol with a commercial kit, the LABSYSTEMS COVID-19 Real Time Multiplex RT-PCR Kit (Labsystems Diagnostics OY, Helsinki, Finland) was used. This kit is designed to detect three genes (ORF1ab, E, N) of the SARS-CoV-2 genome simultaneously. The RT-PCR reactions were set up according to the manufacturer’s protocol and were analysed on a Rotor-Gene Q instrument.

### Calculation of cycle threshold (Ct) and threshold time (Tt) values

The cycle threshold (Ct) values of the RT-PCR assays were calculated from the background corrected amplification curves using the Roto-Gene Q Software [V2.3.5] (Qiagen, Hilden, Germany) or the LightCycler 96 Software [V.1.1.0.1320] (Roche, Basel, Switzerland). Amplification curves from the RT-LAMP were analysed using an identical procedure. As LAMP is an isothermal amplification technique, the reaction time when the fluorescence signal exceeds the threshold is referred to as threshold time (Tt) value.

### Semi-automation of the assays

Semi-automated pipetting of the SARS-CoV-2 RT-LAMP protocol and the in-house RT-PCR were run on a Brand Liquid Handling station (Brand, Wertheim, Germany). The master mixes were prepared manually, and the liquid handler was applied to dispense the master mix into the PCR strips. The isolated RNA samples were collected in 96 well plates and the system was set to transfer an appropriate amount of sample into the prepared PCR strips.

### RT-LAMP performance evaluation

The performance of the SARS-CoV-2 RT-LAMP protocol was assessed by method comparison. To compare the assay with different RT-PCR protocols, 70 RNA isolates from nasopharyngeal swabs which were initially detected as positive (n = 36) and negative (n = 34) for SARS-CoV-2 by in-house RT-PCR were analysed by all three assays and the qualitative results, as well as Ct values of the RT-PCR assays, and the Tt values of the RT-LAMP were compared.

In order to test the semi-automated procedure on the liquid handling station, another 188 RNA isolates were analysed using the in-house RT-PCR protocol and the RT-LAMP assay.

The isolation-free SARS-CoV-2 RT-LAMP test was compared to the in-house RT-PCR using 65 nasopharyngeal swabs. To obtain comparable results, the eluates from these samples were directly aliquoted after elution with NaCl solution (0.9%). One aliquot was analysed with the isolation-free RT-LAMP and a second underwent standard RNA isolation followed by RT-PCR testing using the in-house protocol.

Performance characteristics including limit of detection (LoD), and intra- and inter-run reproducibility of the isolation-free SARS-CoV-2 RT-LAMP protocol were assessed. LoD was determined by serial dilution of a SARS-CoV-2 reference material containing 10^7^ genome copies (INSTAND, Düsseldorf, Germany). Each dilution was analysed in triplicate. To investigate reproducibility of the assay, a negative and a positive sample pool were made from stored SARS-CoV-2 eluates and both were measured in five replicates on three consecutive days.

### Statistical analysis

Positive/negative classification of the Tt values and Ct values from different methods was done by using standardised negative cut-offs. For the RT-PCR assays, a cut-off of Ct 40 was applied and for the RT-LAMP test a cut-off of Tt 25. For data visualization, Ct values and Tt values above these cut-offs were artificially assigned to negative samples (Ct 46 for the RT-PCRs and Tt 31 for the RT-LAMP). Qualitative results were analysed by McNemar’s test and Cohen’s kappa (interrater agreement statistics) for method comparison^46^. Ct values of the different PCR methods were compared with RT-LAMP Tt values by Spearman correlation after testing for normal distribution by applying the Shapiro–Wilk normality test. Statistical testing and data visualization was performed using R [v3.6.3] (R Foundation for Statistical Computing, Vienna, Austria).

## Supplementary Information

Supplementary Information.

## Data Availability

The raw datasets generated during the current study are available from the corresponding author on reasonable request in aggregated form. All data analysed during this study are included in this published article and its Supplementary Information files.
