# Supplementary material for: A semi-automated, isolation-free, high-throughput SARS-CoV-2 reverse transcriptase (RT) loop-mediated isothermal amplification (LAMP) test
Source: Sci Rep. 2021 Nov 1;11:21385. doi: 10.1038/s41598-021-00827-0 (PMC8560768; doi:10.1038/s41598-021-00827-0)
Supplement: Supplementary file 1 — Supplementary Information. [file 41598_2021_827_MOESM1_ESM.docx]

**Supplementary Information**

**A semi-automated, isolation-free, high-throughput SARS-CoV-2 reverse transcriptase (RT) loop-mediated isothermal amplification (LAMP) test**

**Jonas Schmidt, Sandro Berghaus, Frithjof Blessing, Folker Wenzel, Holger Herbeck, Josef Blessing, Peter Schierack, Stefan Rödiger, Dirk Roggenbuck**

Supplement 1: Primer sequences and temperature profile of the in-house SARS-CoV-2 RT-PCR protocol

SARS-CoV-2 E gene PCR Primers (5’ - 3’):

Forward: ACAGGTACGTTAATAGTTAATAGCG

Reverse: TATTGCAGCAGTACGCACAC

SARS-CoV-2 E gene hydrolysis probe (5’ FAM - 3’ BBQ):

ACACTAGCCATCCTTACTGCGCTTCG

SARS-CoV-2 RT-PCR temperature profile:

| Step | Time [min] | Temperature [°C] | Cycle |
| --- | --- | --- | --- |
| Reverse Transcription | 10:00 | 55 | - |
| Initial Denaturation | 3:00 | 94 | - |
| Denaturation | 00:15 | 94 | 45 |
| Annealing | 00:30 | 58 |  |


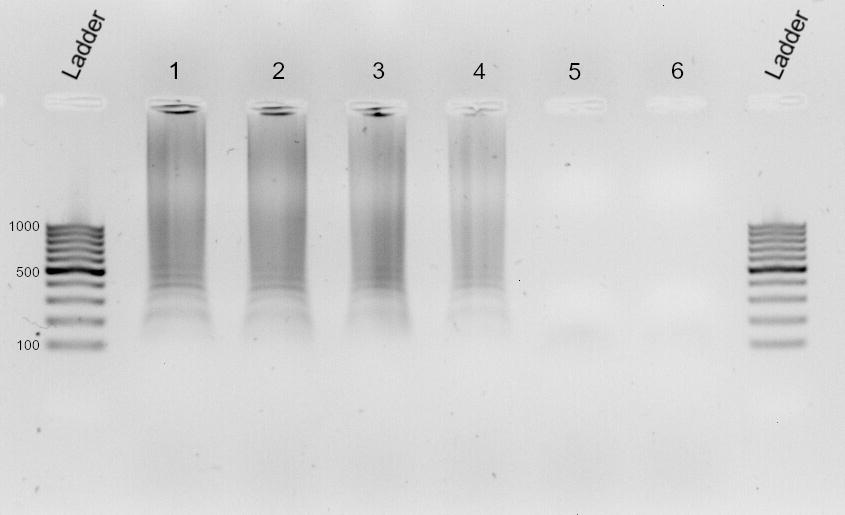


Supplementary Figure S1: Full-length conventional gel electrophoresis of the isolation-free SARS-CoV-2 RT-LAMP reaction products. Reaction products from reference samples containing 10^7^ (1,2) and 10^6^ (3,4) genome copies/ml as well as two no template controls (5,6) were loaded on a 1.5 % agarose gel stained with ethidium bromide after 1:10 dilution with loading dye. The GeneRuler 100 bp DNA Ladder (ThermoFisher Scientific, Waltham, USA) was used as reference. Colour was inverted to improve readability and labels were added using GIMP (v2.10.4). All positive samples show a LAMP characteristic banding pattern. However, this detection method is not recommended for clinical diagnostic laboratories due to the high risk of contamination when opening reaction vessels after successful LAMP reaction.


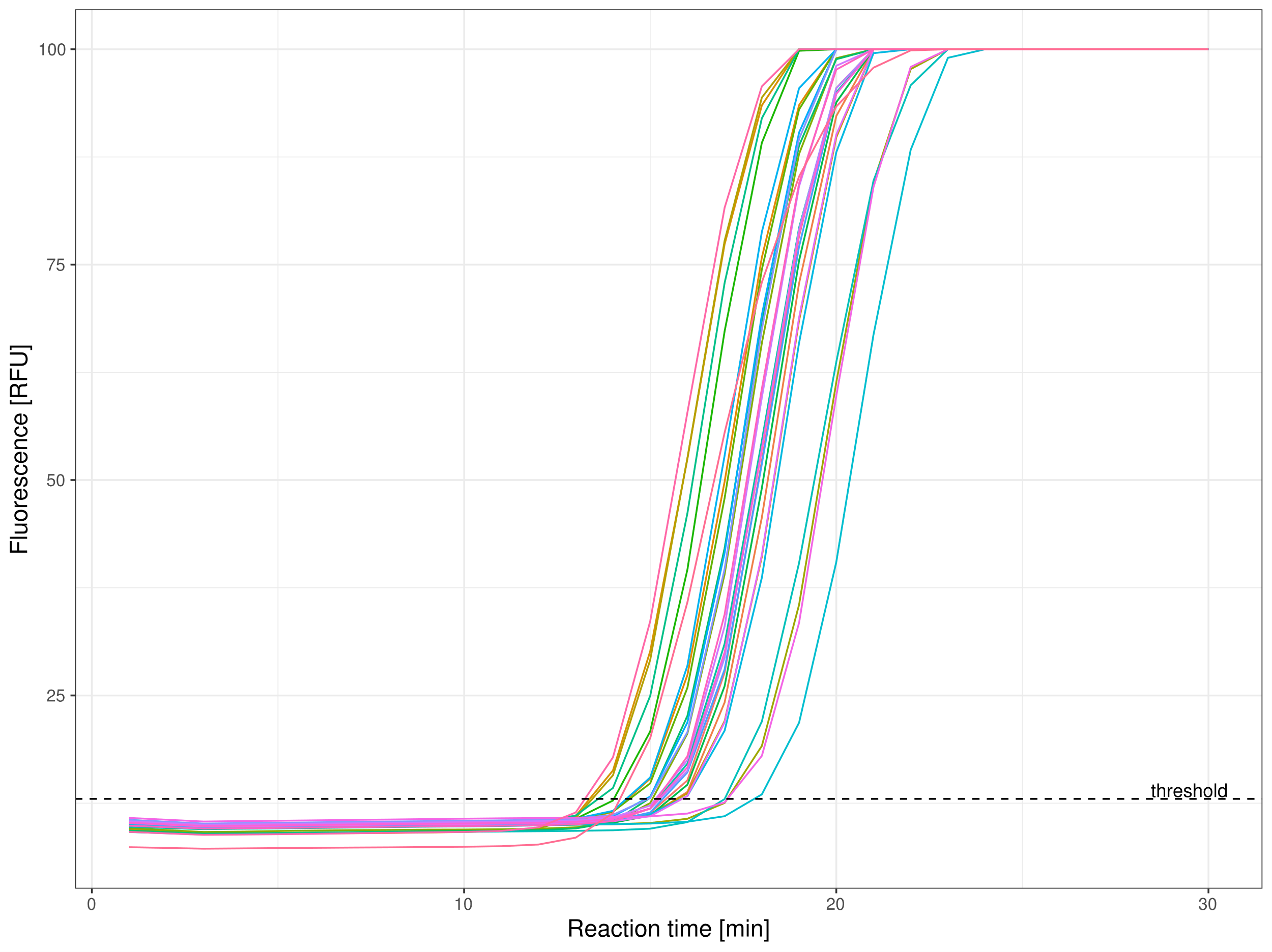


Supplementary Figure S2: Amplification curves of the external control. During initial method establishment, a separate RT-LAMP reaction mix with a primer set targeting human beta actin was set up per sample to account for sample quality. The method comparison experiments were performed without external control to increase throughput and efficiency.

Supplementary Table S1: Limit of detection (LOD) of the isolation-free SARS-CoV-2 RT-LAMP. A serial dilution of a reference sample (10^7^ SARS-CoV-2 copies/ml) was measured in triplicates. The LOD was identified to be at 100,000 copies/ml which is equal to 5,000 copies in the eluate from a nasopharyngeal swap or 95 copies per SARS-CoV-2 RT-LAMP reaction.

| **SARS-CoV-2 genome copies/ml** | **Copy count (elution)** | **Copy count (reaction)** | **Result Run 1 (Tt value) ^[a]^** | **Result Run 2 (Tt value) ^[a]^** | **Result Run 3 (Tt value) ^[a]^** |
| --- | --- | --- | --- | --- | --- |
| 500000 | 25000 | 476 | positive (15.34) | positive  (15.47) | positive  (15.14) |
| 200000 | 10000 | 190 | positive (16.42) | positive  (15.96) | positive  (15.71) |
| 100000 | 5000 | 95 | positive  (16.45) | positive  (17.36) | positive  (16.37) |
| 50000 | 2500 | 48 | positive  (18.25) | positive  (19.6) | negative |
| 25000 | 1250 | 24 | negative | positive  (18.2) | positive  (23.31) |
| 12500 | 625 | 12 | negative | negative | positive  (17.86) |
| 10000 | 500 | 10 | negative | negative | negative |
| 1000 | 50 | 1 | negative | negative | negative |
| 100 | 5 | 0 | negative | negative | negative |

[a] Tt values are only given for positive results.

Supplementary Table S2: Primer sequences of the LAMP primers targeting the E and N gene of SARS-CoV-2. The primer set was prepared as a 10x mix in nuclease free water.

| **Primer** | **Target** | **Sequence 5’-3’** | **Length** | **Concentration 10x Primer Mix [µmol/l]** |
| --- | --- | --- | --- | --- |
| E1-F3 | E gene | TGAGTACGAACTTATGTACTCAT | 23 | 2 |
| E1-B3 | E gene | TTCAGATTTTTAACACGAGAGT | 22 | 2 |
| E1-FIP | E gene | ACCACGAAAGCAAGAAAAAGAAGTTCGTTTCGGAAGAGACAG | 42 | 16 |
| E1-BIP | E gene | TTGCTAGTTACACTAGCCATCCTTAGGTTTTACAAGACTCACGT | 44 | 16 |
| E1-LF | E gene | CGCTATTAACTATTAACG | 18 | 4 |
| E1-LB | E gene | GCGCTTCGATTGTGTGCGT | 19 | 4 |
| N2-F3 | N gene | ACCAGGAACTAATCAGACAAG | 21 | 2 |
| N2-B3 | N gene | GACTTGATCTTTGAAATTTGGATCT | 25 | 2 |
| N2-FIP | N gene | TTCCGAAGAACGCTGAAGCGGAACTGATTACAAACATTGGCC | 42 | 16 |
| N2-BIP | N gene | CGCATTGGCATGGAAGTCACAATTTGATGGCACCTGTGTA | 40 | 16 |
| N2-LF | N gene | GGGGGCAAATTGTGCAATTTG | 21 | 4 |
| N2-LB | N gene | CTTCGGGAACGTGGTTGACC | 20 | 4 |

Supplementary Table S3: Primer sequences of the LAMP primers targeting the human beta actin gene. The primer set was prepared as a 10x mix in nuclease free water. It was used as an external control in a separate reaction set-up per sample during method establishment.

| **Primer** | **Target** | **Sequence 5’-3’** | **Length** | **Concentration 10x Primer Mix [µmol/l]** |
| --- | --- | --- | --- | --- |
| ACTB-F3 | ACTB | AGTACCCCATCGAGCACG | 18 | 2 |
| ACTB-B3 | ACTB | AGCCTGGATAGCAACGTACA | 20 | 2 |
| ACTB-FIP | ACTB | GAGCCACACGCAGCTCATTGTATCACCAACTGGGACGACA | 40 | 16 |
| ACTB-BIP | ACTB | CTGAACCCCAAGGCCAACCGGCTGGGGTGTTGAAGGTC | 38 | 16 |
| ACTB-LF | ACTB | TGTGGTGCCAGATTTTCTCCA | 21 | 4 |
| ACTB-LB | ACTB | CGAGAAGATGACCCAGATCATGT | 23 | 4 |
